# Draft Genome Sequences of Cyclodextrin-Producing Alkaliphilic *Bacillus* Strains JCM 19045, JCM 19046, and JCM 19047

**DOI:** 10.1128/genomeA.00211-14

**Published:** 2014-03-20

**Authors:** Toshiaki Kudo, Kazuaki Sakamoto, Masashi Akinaga, Ayumi Kawauchi, Tomomi Nakahara, Xiaochi Zhang, Akinori Yamada, Kenshiro Oshima, Wataru Suda, Hirokazu Kuwahara, Nobuyuki Nakamura, Yuichi Nogi, Keiko Kitamura, Masahiro Yuki, Toshiya Iida, Shigeharu Moriya, Tetsushi Inoue, Yuichi Hongoh, Masahira Hattori, Moriya Ohkuma

**Affiliations:** a Graduate School of Fisheries Science and Environmental Studies, Nagasaki University, Nagasaki, Japan; b Graduate School of Science and Technology, Nagasaki University, Nagasaki, Japan; c Center for Omics and Bioinformatics, Graduate School of Frontier Sciences, the University of Tokyo, Kashiwa, Japan; d Institute of Biogeosciences, Japan Agency for Marine-Earth Science and Technology (JAMSTEC), Yokosuka, Japan; e Department of Biological Sciences, Graduate School of Bioscience and Biotechnology, Tokyo Institute of Technology, Tokyo, Japan; f Japan Collection of Microorganisms/Microbe Division, RIKEN BioResource Center, Tsukuba, Japan; g Biomass Research Platform Team, RIKEN Biomass Engineering Program Cooperation Division, RIKEN Center for Sustainable Resource Science, Tsukuba, Japan; h Research Cluster for Innovation, RIKEN, and Graduate School of Nanobioscience, Yokohama City University, Yokohama, Japan

## Abstract

*Bacillus* strains JCM 19045, JCM 19046, and JCM 19047 are alkaliphiles that produce β-cyclodextrin from starch. They are related to *Bacillus xiaoxiensis* and *Bacillus lehensis*. The genome information for these three strains will be useful for studies of the physiological role of cyclodextrin and cyclodextrin production.

## GENOME ANNOUNCEMENT

Alkaliphilic *Bacillus* strains produce various alkaline enzymes useful for industry, such as cyclomaltodextrin glucanotransferase (CGTase), amylase, cellulase, hemicellulase, and protease (1, 2). CGTases produce cyclodextrins (CDs) from starch. CDs are cyclic oligosaccharides consisting of 6, 7, or 8 glucopyranose units and are useful for various industrial applications. Various alkaliphilic strains of *Bacillus* spp. producing CDs have been isolated and characterized (3, 4). Although a novel starch degradation pathway via CD has been reported in *Klebsiella oxytoca* (5) and archaea (6, 7), there is still uncertainty regarding the production mechanism of CDs in alkaliphilic *Bacillus* and regarding the physiological role of CDs.

Recently, we isolated CD-producing alkaliphilic *Bacillus* spp. from seaweed near Nagasaki waters, Japan, using an alkaline medium (1). *Bacillus* strains G607 and G608 were isolated from the seaweed *Gelidium elegans*, while *Bacillus* strain G664 was isolated from the seaweed *Polyopes affinis*. These three strains showed CGTase activity and produced β-CD from starch. Strains G607, G608, and G664 were deposited at the Japan Collection of Microorganisms (JCM) (http://www.jcm.riken.jp) and are available as strains JCM 19045, JCM 19046, and JCM 19047, respectively.

The genomes of *Bacillus* strains JCM 19045, JCM 19046, and JCM 19047 were sequenced using an Ion Torrent PGM system. The sequence reads of 361,422 for JCM 19045, 395,701 for JCM 19046, and 376,957 for JCM 19047 were assembled using Newbler version 2.8 (Roche) into 62, 95, and 125 contigs, with *N*_50_ lengths of 123,442, 79,497, and 67,632 bp, respectively. These assemblies resulted in draft genome sequences of 4,314,923 bp for JCM 19045, 4,312,535 bp for JCM 19046, and 4,047,212 bp for JCM 19047, with 17×, 16×, and 16× redundancies and G+C contents of 41.0, 41.0, and 40.0%, respectively. A total of 4,678, 4,763, and 4,300 protein-coding genes and 71, 59, and 65 RNA-coding sequences for JCM 19045, JCM 19046, and JCM 19047, respectively, were identified using the RAST server (8).

Phylogenetic analysis based on 16S rRNA gene sequence comparisons revealed that strains JCM 19045 and JCM 19046 are most closely related to the type strain of *Bacillus xiaoxiensis* (9) (sequence identity, 99%). The 16S rRNA gene sequence identity between strains JCM 19045 and JCM 19046 is 99%. Strain JCM 19047 is most closely related to the type strain of *Bacillus lehensis* (10) (sequence identity, 99%). The genome sequencing of strains JCM 19045 and JCM 19046 revealed the presence of a gene cluster comprising the genes for CGTase, three transporter proteins, and cyclomaltodextrinase (CDase). On the other hand, in the genome sequence of strain JCM 19047, the gene for CGTase and the genes for three transporter proteins and CDase are located separately. The draft genome sequence data for these three strains will be useful for elucidating the physiological roles of CD and the production mechanism of CD.

### Nucleotide sequence accession numbers.

The genome sequences of *Bacillus* strains JCM 19045, JCM 19046, and JCM 19047 have been deposited at DDBJ/EMBL/GenBank under accession no. BAWA01000001 to BAWA01000062, BAWB01000001 to BAWB01000095, and BAWC01000001 to BAWC01000125, respectively.
